# Standardization of DNA Residual Quantification Method of Vero Cell Rabies Vaccine for Human Use

**DOI:** 10.2174/1874104501711010066

**Published:** 2017-06-30

**Authors:** Janeth del Carmen Arias Palacios, Carlos Alberto Barrero Barreto, José Salvador Montaña Lara, Ángela María Londoño Navas

**Affiliations:** 1 Grupo GBAI. Biotecnología Ambiental e Industrial. Facultad de Ciencias Pontificia Universidad Javeriana, Bogota, Colombia; 2 FIDIC Laboratorio de Biología Molecular Fundación Instituto de Inmunología de, Bogota, Colombia; 3 Grupo UNIDIA. Unidad de Investigaciones Agropecuarias. Facultad de Ciencias. Pontificia Universidad Javeriana, Bogota, Colombia; 4 Departamento de Microbiología. Carrera de Microbiología Industrial Pontificia Universidad Javeriana, Bogota, Colombia

**Keywords:** Real time PCR, Residual DNA, Vero cells, Antirrabic vaccine, Betapropiolactone

## Abstract

**Objectives:**

Normalize the quantification of residual DNA from Vero cells in the rabies vaccine for use in human VAHV I, by quantitative PCR in real time and the design of primers that amplified, highly repetitive sequences of *Cercopithecus aethiops* and a constitutive gene according to sequences reported in the GenBank and quantifying the residual DNA in the vaccine VAHV I in three consecutive batches according to the standard set by the World Health Organization.

**Methods:**

A real time quantitative method based on SYBR Green chemistry has been applied for the quantification of residual DNA (resDNA) using highly repetitive DNA (Alu) and a housekeeping gene (B-actin) as target sequences.

**Results:**

The sensitivity achieved with this white sequence is within the reported limits and who are between 5 and 50 pg. For real time PCR optimization with Alu-p53, different concentrations of MgCl2 (0.5, 0.75, 1.0, 1.25 and 1.5 mm) in combination with three different concentrations of primers (75, 100 and 150nM) were used. pDNA in concentration of 1x10^7^ copies / ul was used as template. Optimal concentrations were 1.25 mM MgCl2 and 100nM primers. To level of detection of 1.53 ng/ul was found for p53-Alu and Alu-Glob and 0.39 ng/ul for B-actin with gDNA curves.

**Conclusion:**

Quantification of resDNA of vaccine VAHV I with close-ups of B-actin was normalized. Reached a sensitivity of 30 pg of resDNA/dose VAHV I, with close-ups of B-actin. Found, in three consecutive batches, an amount less than 10 ng/dose, these results suggest that the production process ensures vaccine resDNA removal, meeting international requirements for biological products for use in humans that use continuous cell lines for production.

## INTRODUCTION

Rabies is widely spread across the world being responsible for around 50.000 human deaths per year. Preventionand treatment of the disease is available through immunization. Kidney epithelial cells isolated from African green monkey (Vero cells) are the cell line of choice for rabies vaccine production. Vero cell line has also been used for the production of polio, measles and yellow fever vaccines. During the production of rabies vaccine, vero cells are inoculated with rabies virus, followed by a purificaction step that eliminates cell debris, finally resulting in virus concentration and inactivation with ^®^-propiolactone (bpl) [1, 2].

Although most of the cellular debris are removed during the purification steps, complete absence of resDNA from vero cells cannot be guaranteed. Opinions regarding the risk of resDNA in biological products are divided. Some researchers consider the presence as innocuous but for some others, the presence of resDNA might pose the risk of: malignant transformations due to the activation of cellular or viral oncogenes, aberrant gene expression resulting from sequence insertions in genomic control regions and production of infectious viral particles through the insertion of viral DNA [3].

These potential risks have led the World Health Organization (WHO) to regulate the concentration of resDNA in biological products. Previously, maximum amounts of resDNA were set to 100 picograms (pg) per dose, however, the lack of highly sensitive and reproducible techniques added to more studies regarding the effect of resDNA, have resulted in new guidelines setting the limit to 10 nanograms (ng) per dose.

Hibridization and real time PCR are the techniques used for resDNA quantification. Real time PCR offers higher sensitivity, less sample processing time and reduced risk of sample contamination [3, 4].

In this work, a real time quantitative method based on SYBR Green chemistry has been applied for the quantification of residual DNA (resDNA) in an **Anti-rabies vaccine produced in Vero cells (VAHV I), using highly repetitive DNA (Alu-p53, Alu-glob) and a housekeeping gene (B-actin) as target sequences.**

## MATERIALS AND METHOD

### Vero Cell Culture and DNA Extraction

Vero cells were cultured at 37°C to confluency for 4 days in M199 DMEM in 10 ml flasks. Cells were trypsinized and DNA was extracted (*Wizard→ Genomic* DNA *extraction kit*, Promega #A1120). Spectrophotometry (JASCO Corp. V-550) was used to evaluate purity and concentration with 1/100 dilutions. Genomic DNA integrity was assessed with 1% agarose gel electrophoresis. DNA was resuspended using **RNAse and DNAse** free water and stored at 4°C for further assays [5].

### Primer Design

A GenBank search was performed (http://www.ncbi.nlm.nih.gov) to retrieve housekeeping and highly repetitive sequences within the genome of *Cercopithecus aethiops* (African green Monkey) in order to obtain a higher sensitivity. For Alu-p53, the target gene bank access was U38676. For Alu-Glob, AF057392 and for B-actin, M10277.

Once the sequences were selected, Primer 3 output (http://www.broad.mit.edu/cgi-bin/primer/primer3_www.cgi) and VectorNTI were chosen for the design of primer sequences. Primers were synthesized by IDT (*Integrated DNA Technologies, Inc,* Coralville, USA).

### Vero Cell DNA Amplification

Conventional PCR were performed in 25 µl. for the 3 sets of primers. Conditions were as follows: buffer 1X (Promega), MgCl_2_ 3 mM, dNTP´s 0.2 mM, primers 400nM, Taq DNA polimerase 0.625 U and genomic Vero DNA 400 y 40 ng. For all primers tested cycles were set to: 95°C for 5 min, 30 cycles of 95°C for 1 min, 62°C for 1 min and 72°C for 1 min with a final step at 72°C for 5 min. Negative control with DNAse and RNAse free water were used.

A positive control for amplification was used. *Plasmodium vivax* genomic DNA was amplified using MSP-1 region 3 primers with a known product of 300bp. 2% agarose gel was used to visualize PCR products.

### Cloning

PCR fragments were cloned into plasmids using pGEM-T *Easy Vector System* I (Promega #1360) according to the manufacturer’s protocol. After plasmid purification, concentration was adjusted to 100 ng/µl with a 260/280 nm ratio of 1.8. Plasmids were further sequenced. (Bioresource Center, Cornell University, Ithaca NY).

### Plasmid DNA Extraction

Plasmid DNA was extracted using *Wizard*^®^ Plus *Minipreps* DNA *Purification System* (Promega #A7500) according to the manufacturer’s protocol and evaluated by electrophoresis and spectrophotometry. Copy numbers for each of the three inserts were calculated using the following equation [6, 7].


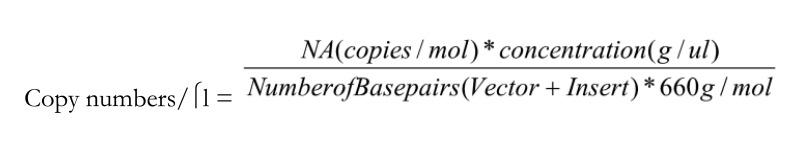


Where, NA represents Avogadro’s number and concentration refers to plasmid DNA concentration. Plasmids were kept at -20°C for further assays.

### Quantitative Real Time PCR Standardization

To avoid formation of primer dimers, concentrations of MgCl_2_ and primers were optimized. RT-PCR reactions were carried out using SYBR^®^ Green PCR core reagents kit (Apllied Biosystems) and GeneAmp 5700 *Sequence Detection System* (Applied Biosystems).

For the RT-PCR reactions, optical tubes were used (ABI PRISM Applied Biosystems, #4323032) and varied concentrations of MgCl_2_ (0.5-2mM) and primers (25-150nM) were tested. Reactions were carried out with a final volume of 20 µl, SYBR Green 1X buffer, 1mM dNTP´s, 0.2 U UNG, 0.5 U AmpliTaq Gold DNA polimerase and 2µl template. DNA amplification program was: 50°C for 2 minutes UNG for UNG activation, followed by 40 cycles at 95°C for 15s, 62°C for 30s and 72°C for 30s. A final dissociation curve protocol was performed to allow identification of amplification products according to melting temperatures T_m_. In all cases, negative controls were used [8].

Standard curves using both genomic and plasmidic DNA as templates were evaluated with the three sets of primers designed. All reactions used a final volume of 20 µl with concentrations of MgCl_2_ and primers according to previous reaction optimization. All remaining reactions included SYBR Green, 1X buffer, 1mM dNTP´s, 0.2 U UNG, 0.5 U AmpliTaq Gold DNA polimerase and 2µl template. Amplification program was the same as described above. For eah set of primers, a standard curve was performed using both gDNA and pDNA and each concentration point was evaluated in triplicate. pDNA concentrations were: 1x10^7^, 4x10^5^, 1.6x10^4^, 6.4x10^2^ y 2.566x10^1^ copies/µl. gDNA concentrations were 100, 25, 6.25, 1.53 y 0.39 ng/µl. Correlation coefficient was used to validate standard curves and the efficiency of reactions was calculated (equation 1). These values should be equal or higher than 0.95 y 90%, respectively.

## EVALUATION OF VAHV I AS A TEMPLATE FOR QUANTITATIVE REAL TIME PCR

VAHV I (batch 005) was resuspended in 500µl of DNases/RNases free water. Half of the dose was mock inoculated with pDNA for a final concentration of 4x10^5^ copies/µl.

Initially, 10µl of theses samples were used as template. For this assay Alu-p53 primers were used and a standard curve with the following pDNA concentrations was done: 1x10^7^, 2x10^6^, 4x10^5^, 8x10^4^, 1.6 x 10^4^, 3.2x10^3^, 6.4x10^2^, 1.28x10^2^ and 2.56x10^1^ copies/µl. VAHV I vaccine contains inactivated viral particles, saccharose and dextran. These components are not considered to be common PCR inhibitors, however it was necessary to evaluate if some components could somehow inhibit RT-PCR reaction. To assess this, different volumes of vaccine template (VAHV I inoculated with 4x10^5^ copies/µl) were used. (2, 4, 6, 8 and 10 µl). Alu-p53 primers were used for these reactions. pDNA (1x10^7^ copies/µl) and gDNA (100 ng/µl) were used as positive controls.

Since concentration of resDNA in VAHV I vaccine is expected to be low, two methods of resDNA reconcentration were used. Isoproponal precipitation and use of Wizard→ Genomic DNA extraction kit (Promega #A1120) omitting cell lysis step. For this purpose, a new dose of VAHV I batch 005 was resuspended in 500µl of DNases/RNases free water. Half of the dose was used for isopropanol precipitation and the other half was used with the extraction kit. Another VAHV I batch 005 was mock inoculated with gDNA for a concentration of 50ng/dose and the DNA extraction kit was used.

Vaccines that were mock inoculated and extracted were evaluated as well as resuspended vaccines without any further treatment. Alu-p53 and B-actin primers were used. Reactions were carried out using a standard curve with pDNA (1x10^7^, 2x10^6^, 4x10^5^, 8x10^4^, 1.6x10^4^, 3.2x10^3^, 6.4x10^2^, 1.28x10^2^ and 2.56x10^1^ copies/µl).

## Residual DNA Quantification of Vero Cells in Vahv I

VAHV I resDNA quantification was evaluated in three consecutive batches (005, 006 and 007) using three doses per batch. To reconcentrated resDNA a Wizard→ Genomic DNA extraction kit was used (Promega #A1120). Anti-rabies vaccine (VAHV II) for human use produced in Vero cells, was used as a reference. Three doses of the same batch were used. All doses of VAHV II were resuspended in DNases/RNAases water. Half the dose was used directly as template and the other half was extracted using the kit mentioned above. Each sample was evaluated in triplicate for each set of primers.

For the quantification of resDNA (ng/dose) in vaccines gDNA curves were used. Validation was done through correlation coefficient and efficiency calculation. gDNA curves were also compared to pDNA curves.

For Alu-p53 primers pDNA dilutions were: 1x10^7^, 2x10^6^, 4x10^5^, 8x10^4^, 3.2x10^3^ and 1.28x10^2^ copies/µl and 100, 50, 25, 12.5, 3.13 and 1.56 ng/µl of gDNA. For Alu-Glob primers pDNA dilutions were: 1x10^7^, 2x10^6^, 4x10^5^, 8x10^4^, 3.2x10^3^, 6.4x10^2^, 1.28x10^2^ and 2.56x10^1^ copies/µl and 100, 50, 3.13 and 1.56 ng/µl of gDNA. For B-actin pDNA dilutions were 1x10^7^, 2x10^6^, 4x10^5^, 8x10^4^, 3.2x10^3^, 6.4x10^2^, 1.28x10^2^ and 2.56x10^1^ copies/µl and 100, 50, 25, 12.5, 3.13, 1.56 0.78 and 0.39ng/µl of gDNA.

## RESULTS AND DISCUSION

### Primer Design

Nucleotide sequence search was performed (http://www.ncbi.nlm.nih.gov). Three sequences were selected for primer design. Two sequences belong to Alu elements from *Cercopithecus aethiops.* The first sequence is located at the intron 6 from tumor suppressor gene p53. The second sequence selected is an intergenic region from psi, beta and delta globin genes. Human beta actin gene was selected as a constitutive gene. Primers were obtained using Vector NTI and Primer 3 Output are shown in (Table **1**).

## Amplification and Cloning of Specific Genomic Sequences From Vero Cells

Genomic DNA from Vero cells was used for conventional PCR with primers designed. Amplification product size with Alu-p53, Alu-Glob and B-actin primers were as expected. Fig. (**1**).

**Fig. (4):**
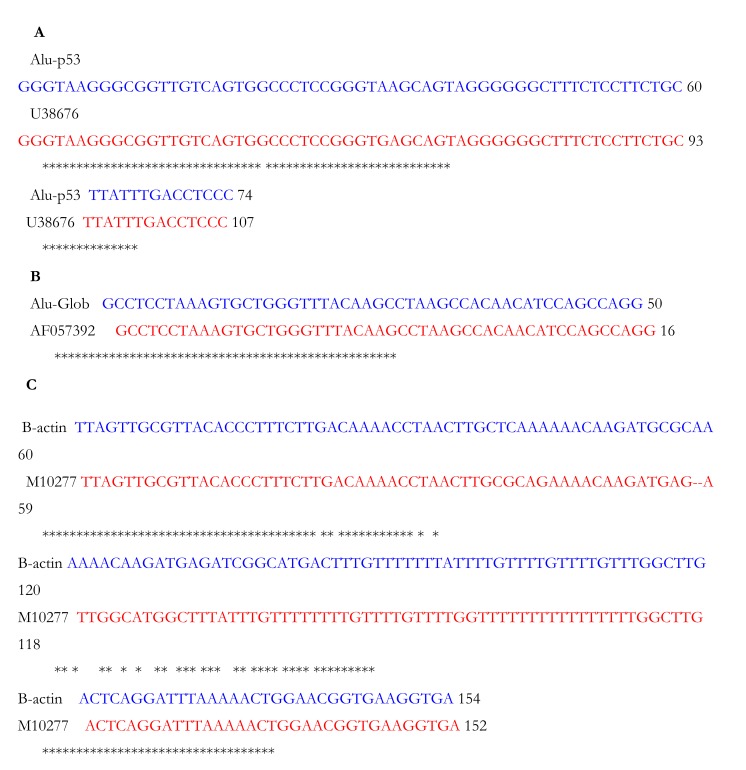
Alignment of sequenced plasmids. In A, B and C. Blue, results from sequencing of Alu-p52, Alu-Glob and B-actin plasmids. In red, partial sequence reported in GenBank.

Gel ligation was performed to avoid problems with unspecific PCR products. Colony PCR was used to select clones Fig. (**2**) that were further sent for sequencing. Fig. (**2** and **3**). CLUSTALW was used to align sequenced clones Alu-p53 1-1, Alu-Glob 2-9 and B-actin 3-11 with homologies to target GenBank sequences of 98, 100 and 80%, respectively [9, 10]. (Fig. **4**).

All standard curves were evaluated for their correlation coefficient and efficiency of the reactions were calculated. (equation 1) Results are shown in (Table **5**).

1E=10-1slope-1

## REAL TIME PCR STANDARDIZATION

### PCR Product Identification Using SYBR Green Dissociation Curves

SYBR Green allows the use of a dissociation protocol that differentiates amplification products according to their melting temperatures (Tm). This is particularly useful to differentiate between primer dimers and specific PCR products. Figs. (**5**, **6** and **7**) show amplification peaks for the PCR products with the three primers selected using both gDNA and pDNA as template. For Alu-p53, Alu-Glob and B-actin product Tm were 82, 78.8 and 78.4°C, respectively. Peaks shown for the non template control (NTC) was attributed to primer dimer formation.

### Real Time PCR Optimization

For real time PCR optimization with Alu-p53, different concentrations of MgCl_2_ (0.5, 0.75, 1.0, 1.25 and 1.5mM) in combination with three different concentrations of primers (75, 100 and 150nM) were used. pDNA in a concentration of 1x10^7^ copies/µl was used as template. Optimal concentrations were 1.25mM MgCl_2_ and 100nM primers [2].

RT PCR standardization for Alu-p53, Alu-Glob, and B-Actin and the effect of MgCl2 and primers on Ct. The results showed in (Tables **2**, **3** and **4**). Respective combinations of MgCl_2_ and primers are shown in Tables for the remaining primers used. Optimal conditions for Alu-Glob were 1.25 mM de MgCl_2_ and 100nM. For B-actin 2mM MgCl_2_ and 50nM primers were selected initially, however after evaluation of the standard curve and the efficiency results concentration of MgCl_2_ was increased to **75mM.**

Different efficiencies and levels of detection were obtained for the three sets of primers using gDNA or pDNA as template Table (**7**). For Alu-p53 the dilution containing 2.56x10^1^copies/µl Fig. (**8B**) showed a significant primer dimer formation. For this reason, this point was remove from the curve. A level of detection of 0.39 ng/µl using gDNA as template was obtained Fig. (**8B**). Reaction efficiencies were 90% or higher for gDNA and pDNA curves.

**Fig. (9):**
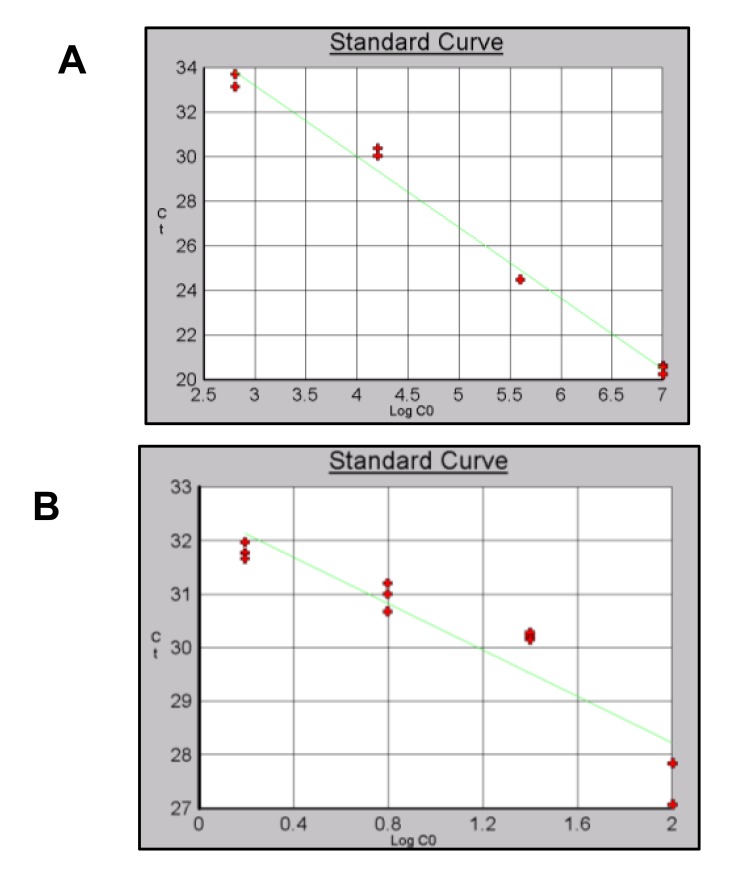
Standard curves using pDNA and gDNA as template and Alu-Glob primers. A) pDNA B) gDNA.

A level of detection of 6.40 x10^2^ copies/µl and 1.56ng/µl was obtained for Alu-Glob (Figs. **9A** and **B**).

**Fig. (10):**
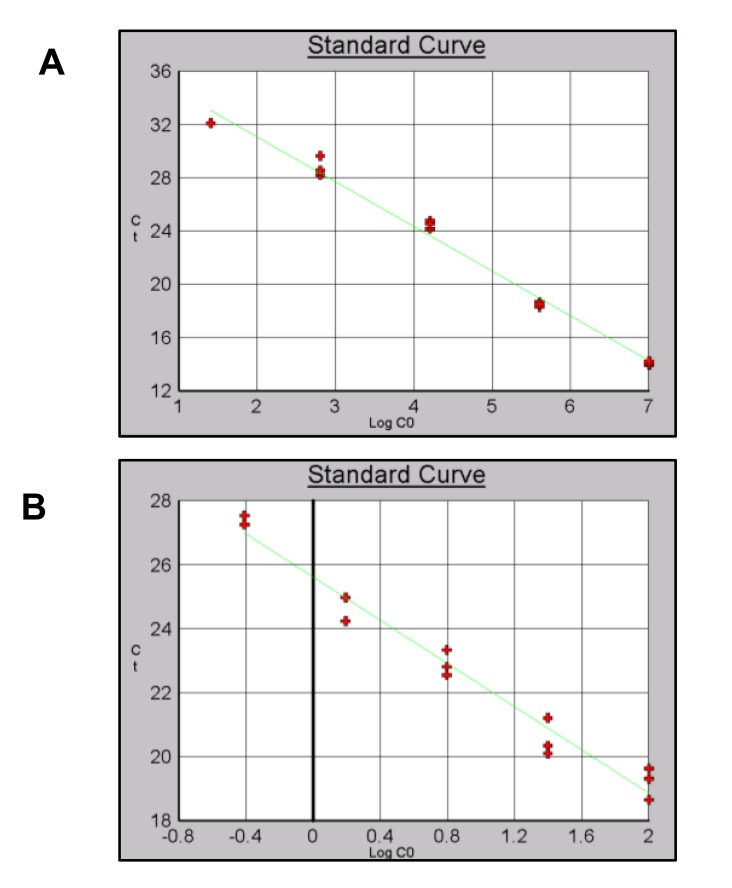
Standard curves using pDNA and gDNA as template and B-actin primers. A) pDNA B) gDNA.

Finally for B-actin a level of detection of 2.56x10^1^ copies/µl for pDNA and 0.39 ng/µl for gDNA was obtained with and efficiency of 98% (Figs. **10A** and **B**).

## EVALUATION OF VAHV I AS TEMPLATE FOR QUANTITATIVE REAL TIME PCR

VAHV I batch 005 vaccine, with or without addition of pDNA did not show amplification using 10 µl as template as shown in Fig. (**11**). To evaluate possible template inhibition, different volumes of template were used (2, 4, 6, 8 and 10 µl) to dilute potential inhibitors.

10^7^

The presence of potential inhibitors was confirmed. Using 2µl of template resulted in product amplification with a vaccine containing pDNA. However, VAHV I without DNA addition failed to amplify with any volume used as observed in (Table **6**).

Resuspended vaccine samples from batch 005 were further DNA extracted to discard presence of resDNA as previously described. Samples were amplified using Alu-p53 and B-actin primers. According to the results VAHV I has some presence of resDNA and kit extraction was significantly more efficient than isopropanol precipitation.

## RESIDUAL DNA CUANTIFICATION OF VERO CELLS IN VAHV I

For resDNA quantification standard curve with pDNA and gDNA were used. pDNA curves were used as an additional validation parameter. Alu-p53 based curve was the least sensitive, detecting 1.28x10^2^ copies/µl. A level of detection of 2.56x10^1^ copies/µl was obtained with Alu-Glob and B-actin curves.

A level of detection of 1.53 ng/µl was found for Alu-p53 and Alu-Glob and 0.39 ng/µl for B-actin with gDNA curves. Correlation coefficient and efficiencies for all standard curves were higher than 0.97 and 90% respectively and identification of product specificity was assessed through dissociation curves.

To calculate resDNA from VAHV I and VAHV II an average of the Ct’s was used. Total residual DNA quantification varied according to primers used. Furthermore, total resDNA varied among batches and samples from the same batch. The highest level of resDNA was found with Alu-p53 primers followed by Alu-Glob. A level of 7.7µg de resDNA/dose was found for a sample of VAHV IIB vaccines. A sample of VAHV I batch 006b using Alu-p53 and Alu-Glob primers detected 112 and 429ng per dose respectively.

Finally with B-actin primers resDNA quantification did not exceed 9 ng/dose for all samples, detecting levels of down to 30 pg de resDNA/dose for VAHV I batch 006b. Using this set of primers only sample VAHV II (VAHV IIC) amplified with a level of resDNA of 5.5ng/dose Table (**7**). The results obtainted with VAHVII using B-actin primers suggest that can be consider as a good method for Vero cells resDNA quantification. In the other hand, the levels detected in VAHVI indicate a production process that ensures a greater removal of resDNA.

## CONCLUSION

In this work, three sets of primers were designed to allow further quantification of resDNA in rabies human vaccine to meet the WHO requirement of less than 10ng/dose. Selection of highly repetitive sequences might overestimate the quantity of resDNA. Results obtained with B-actin primers are consistent with other reports which have previously used this sequence for the resDNA quantification in other biological products that use primate cell lines as substrate.

It was possible to normalize resDNA quantification for VAHV I vaccine with B-actin primers in three consecutive batches finding less than 10 ng/dose of resDNA suggesting that production process insures adequate removal of resDNA complying with international requirements for biological products for human use.

In order to improve the quantification of DNA in these vaccines using alu-p53 primers, it is necessary to treat previously the gDNA samples with compounds that reduce the secondary structure in order to increase the annealing efficiency of the primers.

## Figures and Tables

**Fig. (1):**
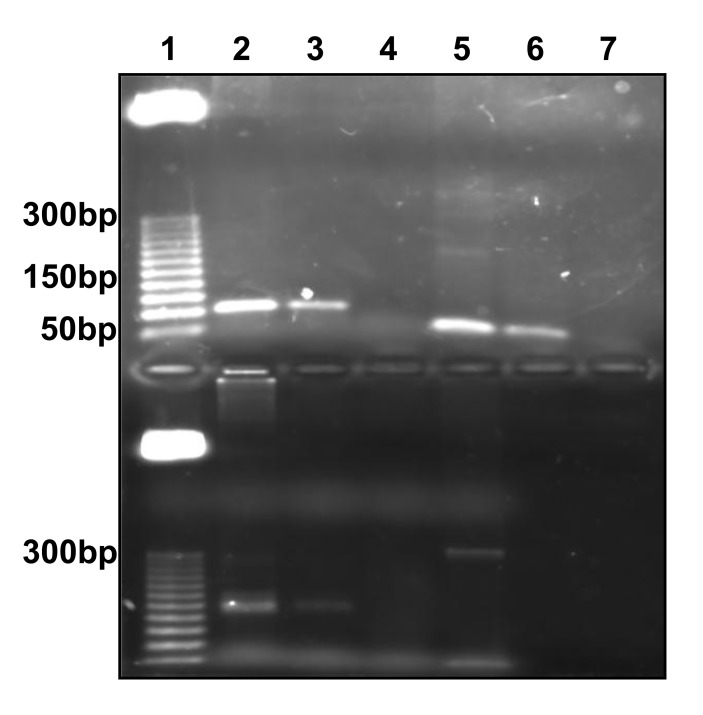
PCR from genomic Vero cell’s DNA. Top gel. Lane 1, MPM 25bp (**Promega).** Lanes 2 and 3, amplification product with Alu-p53 primers with 400 and 40ng DNA. Lane 4, negative control. Lanes 5 and 6, amplification product with Alu-Glob primers with 400 and 40ng DNA. Lane 7, negative control. Bottom gel. Lane 1, MPM 25bp (**Promega)**. Lanes 2 and 3, amplification product with B-actin primers with 400 y 40ng DNA. Lane 4, negative control. Lane 5, amplification product with MSP-1 region 3 primers with *Plasmodium vivax*

**Fig. (2):**
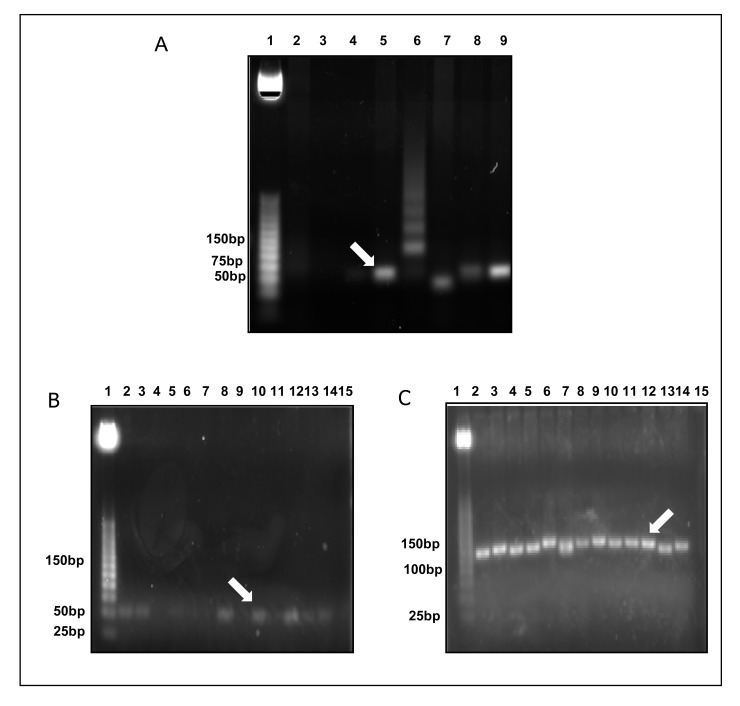
Colony PCR A) Lane 5, clone Alu-p53 1-1. B) Lane 10, clone Alu-Glob 2-9. C) Lane 12, clone B-actin 3-11. Lane 1, A, B and C. MPM 25bp. **(Promega)** 2% agarose gel

**Fig. (3):**
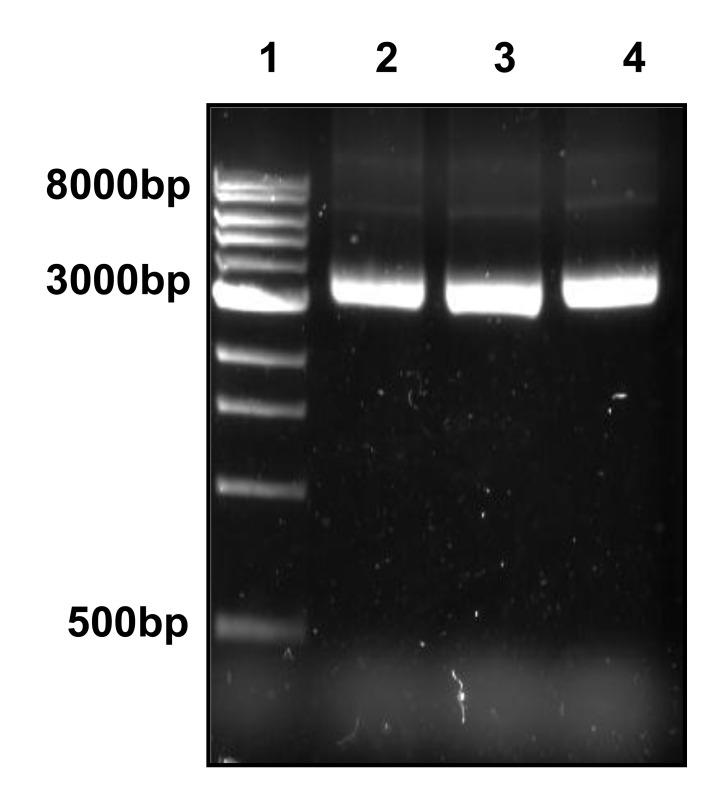
Plasmidic DNA extraction. Lane 1, MPM 1kb (**Ladder Promega**); Lane 2-4, plasmidic DNA Alu-p53 1-1, Alu-Glob 2-9 and B-actin 3-11. 1% agarose gel.

**Fig. (5):**
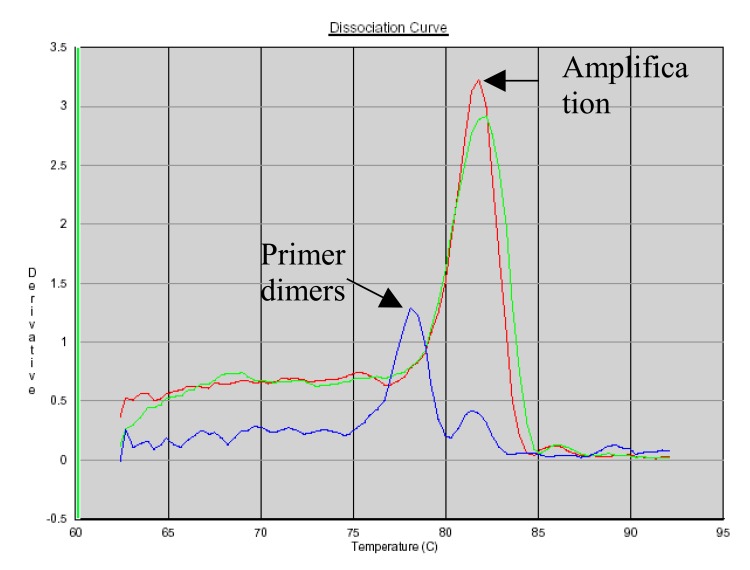
Dissociation curves of amplification products with Alu-p53 primers. Red, pDNA as template; green, gDNA as template; blue, NTC.

**Fig. (6):**
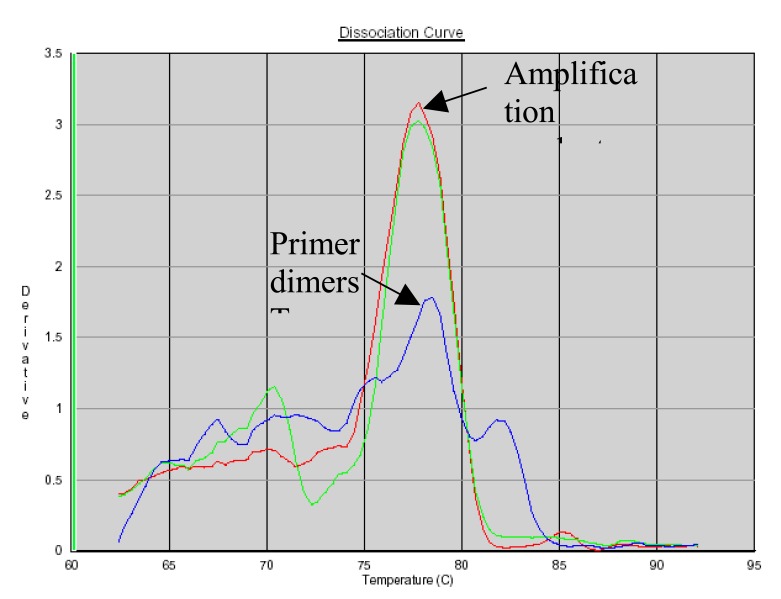
Dissociation curves of amplification products with Alu-Glob primers. Red, pDNA as template; green, gDNA as template; blue, NTC.

**Fig. (7):**
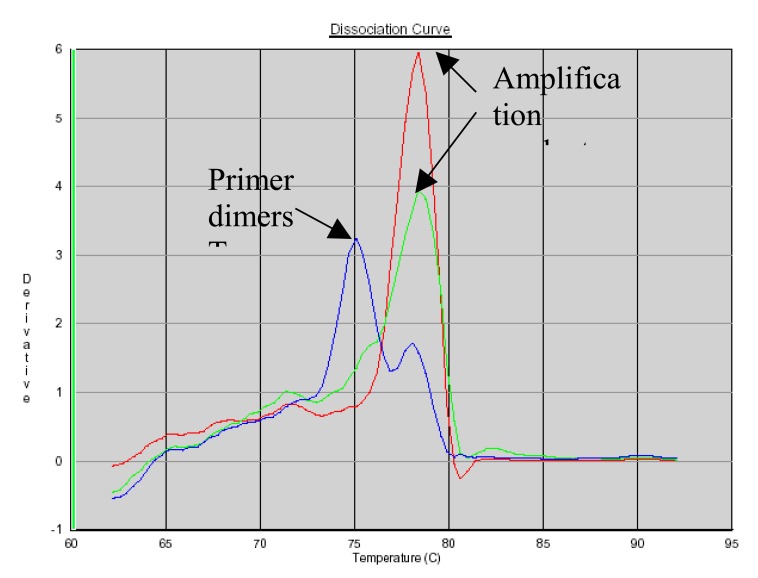
Dissociation curves of amplification products with B-actin primers. Red, pDNA as template; green, gDNA as template; blue, NTC.

**Fig. (8):**
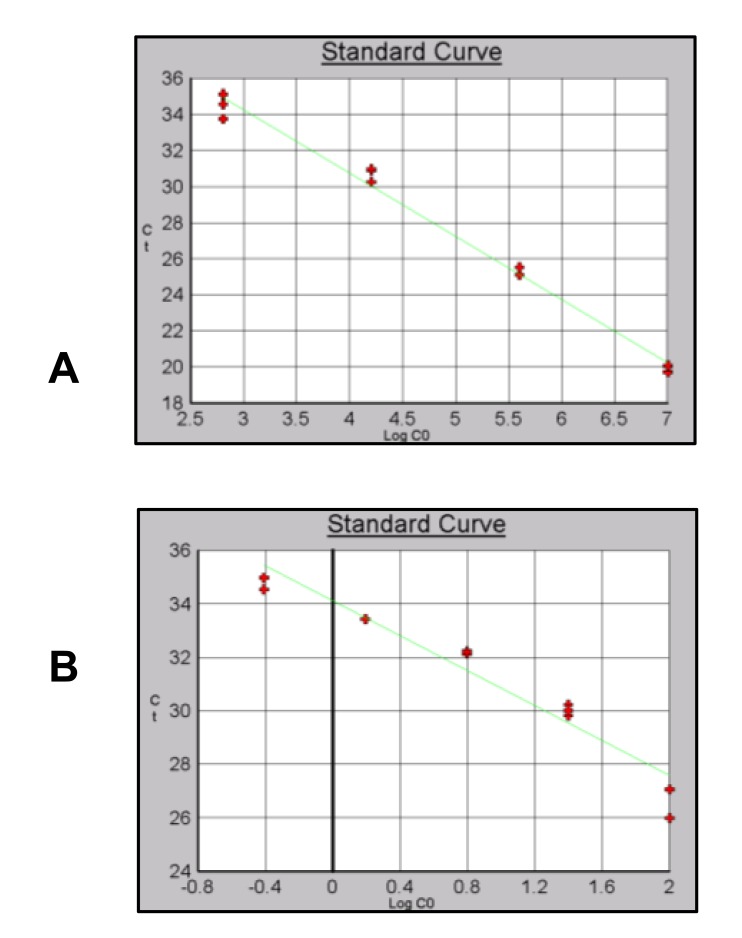
Standard curves using pDNA and gDNA as template and Alu-p53 primers. A) pDNA) B) gDNA.

**Fig. (11):**
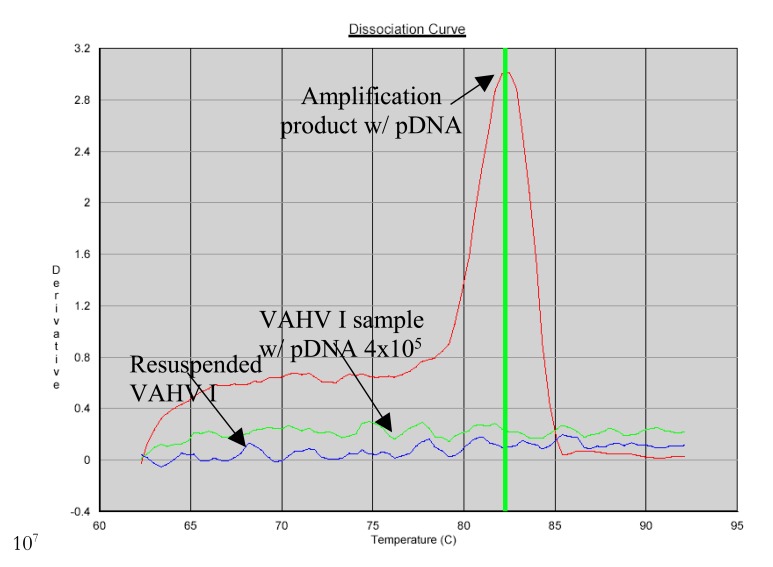
RT-PCR reaction with 10µl of VAHV I as template.

**Table 1:** Selected primers for real time PCR.

**Primers**	**Sequence** **(5´-3´)**	**Tm**	**%GC**	**PCR product (bp)**	**Target sequence.** **Access Number GenBank**
5´ Alu-p53	GGG TAA GGG CGG TTG TCA GT	63.5	60	74	U38676
3´Alu-p53	GGG AGG TCA AAT AAG CAG AAG GAG	63.6	50		
5´ Alu-Glob	CCT GGC TGG ATG TTG TGG	61.1	61.1	50	AF057392
3´ Alu-Glob	GCC TCC TAA AGT GCT GGG TT	61.8	52.3		
5´ B-actin	TTA GTT GCG TTA CAC CCT TTC TTG	61.7	41.6	152	M10277
3´ B-actin	TCA CCT TCA CCG TTC CAG TTT	61.8	47.6		

**Table 2:** RT-PCR standardization for Alu-p53. Effect of the concentrations of MgCl2 and primers on Ct (threshold cycle, defined as the number of cycles required for the fluorescent signal to cross the threshold).

**Template**	**[MgCl_2_]**	**[*primers*]**	**Ct**
pDNA 1 x10^7^ copies/µl	1.5mM	150 nM	11,63
		100 nM	12,75
		75 nM	14,99
NTC		150 nM	35,42
		100 nM	36,36
		75 nM	37,07
pDNA 1 x10^7^ copies/µl	1.25mM	150 nM	13,4
		100 nM	18,53
		75 nM	19,06
NTC		150 nM	37,52
		100 nM	40
		75 nM	40
pDNA 1 x10^7^copies/µl	1mM	150 nM	30,54
		100 nM	40
		75 nM	40
NTC		150 nM	40
		100 nM	40
		75 nM	40
pDNA 1 x10^7^ copies/µl	0.75mM	150 nM	40
		100 nM	40
		75 nM	40
NTC		150 nM	40
		100 nM	40
		75 nM	40
pDNA 1 x10^7^copies/µl	0.5mM	150 nM	40
		100 nM	40
		75 nM	40
NTC		150 nM	40
		100 nM	40
		75 nM	40

**Table 3:** RT-PCR standardization for Alu-Glob. Effect of the concentration of MgCl2 and primers on Ct.

**Template**	**[MgCl_2_] and [*primers*]**	**Ct**
pDNA 1 x10^7^ Copies/µl	1.75mM - 50 nM	17,87
gDNA Vero 50ng/µl		27,36
NTC		36,68
pDNA 1 x10^7^ Copies/µl	1.5mM - 50 nM	21,29
gDNA Vero 50ng/µl		30,98
NTC		40
pDNA 1 x10^7^ Copies/µl	1.25mM – 75 nM	22,65
gDNA Vero 50ng/µl		32,33
NTC		40
pDNA 1 x10^7^ Copies/µl	1.25mM – 100 nM	19,92
gDNA Vero 50ng/µl		25,01
NTC		40

**Table 4:** RT-PCR standardization for B-actin. Effect of the concentration of MgCl_2_ and primers on Ct.

**Template**	**[MgCl_2_]**	**[*Primers*]**	**Ct**
pDNA 1 x10^7^ Copies/µl	2mM	25	37,02
gDNA Vero 50ng/µl			38,66
NTC			39,95
pDNA 1 x10^7^ Copies/µl		40	22,27
gDNA Vero 50ng/µl			29,72
NTC			40
pDNA 1 x10^7^Copies/µl		50	17,29
gDNA Vero 50ng/µl			24,16
NTC			27,41*
pDNA 1 x10^7^ Copies/µl		75	16,46
gDNA Vero 50ng/µl			24,13
NTC			40
pDNA 1 x10^7^ Copies/µl	1.75mM	25	40
gDNA Vero 50ng/µl			40
NTC			40
pDNA 1 x10^7^Copies/µl		40	26,17
gDNA Vero 50ng/µl			35,82
NTC			40
pDNA 1 x10^7^ Copies/µl		50	40
gDNA Vero 50ng/µl			40
NTC			40
pDNA 1 x10^7^Copies/µl		75	21,57
gDNA Vero 50ng/µl			28
NTC			33,69
pDNA 1 x10^7^ copies/µl	1.5mM	25	40
gDNA Vero 50ng/µl			38,56
NTC			40
pDNA 1 x10^7^ copies/µl		40	35,9
gDNA Vero 50ng/µl			34,8
NTC			40
pDNA 1 x10^7^ copies/µl		50	31,07
gDNA Vero 50ng/µl			38,59
NTC			40
pDNA 1 x10^7^ copies/µl		75	35,54
gDNA Vero 50ng/µl			35,96
NTC			37,87

* The signal observed for NTC template correspond to *primers* dimers and was not observed in subsequent assays.

**Table 5:** Efficiency comparison of standard curves with three sets of primers.

**Template for standard curves**	**Slope**	**Efficiency %**	**Correlation coefficient**
Alu-p53 pDNA	-3,51	93	0,9948
Alu-p53 gDNA	-3,26	102	0,9628
Alu-Glob pDNA	-3,19	106	0,9949
Alu-Glob gDNA	-2.16	190	0,9267
B-actin pDNA *primers* 50nM	-3.04	113	0.9718
B-actin gDNA *primers* 50nM	-4.96	59	0,9469
B-actin pDNA *primers* 75nM	-3,37	98	0,9941
B-actin gDNA *primers* 75nM	-3,38	98	0,9850

**Table 6:** Quantification of resDNA in VAHV I and VAHV II vaccines with B-actin primers with RT-PCR.

Samples	Ct	Initial [resDNA] ng/µl	ng of resDNA/dose
VAHV I L005a	35.35	0.0069	0.345
VAHV I L005b	30.51	0.1761	8.807
VAHV I L005c	36.25	0.0038	0.189
VAHV I L006a	36.51	0.0032	0.159
VAHV I L006b	38.98	0.0006	0.030
VAHV I L006c	37.87	0.0013	0.064
VAHV I L007a	40	--	--
VAHV I L007b	32.01	0.0645	3.226
VAHV I L007c	37.36	0.0018	0.090
VAHV IIA	40	--	--
VAHV IIA w/out extraction	40	--	--
VAHV IIB	40	--	--
VAHV IIB w/out extraction	40	--	--
VAHV IIC	32.24	0.06	5.531
VAHV IIC w/out extraction	40	--	--
NTC	40	--	--

**Table 7:** Effect of VAHV I volume template on amplification (Ct).

Sample	Volume of template added (ul)	Ct
pDNA 1x10^7^	2	21.24
gDNA 100ng/µl	2	35.41
VAHV I 4x10^5^ copies/µl	2	35.01
	4	40
	6	40
	8	40
	10	40
VAHV I W/OUT DNA	2	40
	4	40
	6	40
	8	40
	10	40
NTC	0	40
